# Evaluation of consensus method for the culture of *Burkholderia pseudomallei* in soil samples from Laos

**DOI:** 10.12688/wellcomeopenres.14851.2

**Published:** 2018-11-21

**Authors:** David A.B. Dance, Michael Knappik, Sabine Dittrich, Viengmon Davong, Joy Silisouk, Manivanh Vongsouvath, Sayaphet Rattanavong, Alain Pierret, Paul N. Newton, Premjit Amornchai, Vanaporn Wuthiekanun, Sayan Langla, Direk Limmathurotsakul

**Affiliations:** 1 Lao-Oxford-Mahosot Hospital-Wellcome Trust Research Unit, Mahosot Hospital, Vientiane, Lao People's Democratic Republic; 2 Centre for Tropical Medicine and Global Health, University of Oxford, Oxford, OX3 7FZ, UK; 3 Faculty of Infectious and Tropical Diseases, London School of Hygiene & Tropical Medicine, London, WC1E 7HT, UK; 4 Médecins Sans Frontières, Maputo, Mozambique; 5 Foundation for Innovative Diagnostics, Geneva, Switzerland; 6 Institut de Recherche pour le Développement (IRD), iEES-Paris (IRD, Sorbonne Universités, UPMC Univ Paris 06, CNRS, INRA, UPEC, Université Paris Diderot), Department of Agricultural Land Management (DALaM), Vientiane, Lao People's Democratic Republic; 7 Mahidol-Oxford Tropical Medicine Research Unit, Faculty of Tropical Medicine, Mahidol University, Bangkok, 10400, Thailand

**Keywords:** Burkholderia pseudomallei, melioidosis, soil, environmental samples, culture, detection, Laos, Lao PDR

## Abstract

**Background:** We have previously shown that PCR following enrichment culture is the most sensitive method to detect *Burkholderia pseudomallei* in environmental samples. Here we report an evaluation of the published consensus method for the culture of *B. pseudomallei* from Lao soil in comparison with our conventional culture method and with PCR with or without prior broth enrichment.

**Methods:** One hundred soil samples were collected from a field known to contain *B. pseudomallei *and processed by: (i) the conventional method, (ii-iii) the consensus method using media prepared in either Laos or Thailand, and (iv) the consensus method performed in Thailand, as well as by (v) PCR following direct extraction of DNA from soil and (vi) PCR following broth pre-enrichment.

**Results:** The numbers of samples in which *B. pseudomallei* was detected were 42, 10, 7, 6, 6 and 84, respectively. However, two samples were positive by the consensus method but negative by conventional culture, and one sample was negative by PCR following enrichment although *B. pseudomallei* was isolated by the conventional culture method.

**Conclusions/Discussion:** The results show that no single method will detect all environmental samples that contain *B. pseudomallei.* People conducting environmental surveys for this organism should be aware of the possibility of false-negative results using the consensus culture method. An approach that entails screening using PCR after enrichment, followed by the evaluation of a range of different culture methods on PCR-positive samples to determine which works best in each setting, is recommended.

## Introduction

Melioidosis, or infection with *Burkholderia pseudomallei*, is an important but under-recognised public health problem throughout the tropics ^1^. The causative agent is a Gram-negative saprophyte found in soil and surface water in endemic areas. There have been numerous studies describing the detection of *B. pseudomallei* from soil ^2– 31^. These studies have used a wide range of both culture and molecular approaches. In 2013, an attempt was made to standardise these approaches, and a culture method, based on a technique that had a comparable sensitivity to semiquantitive culture on solid media during a small-scale evaluation in northeast Thailand ^32^, was published and proposed as a consensus methodology ^33^. This method, which uses enrichment culture and is thus only qualitative, has not yet been formally evaluated elsewhere. During studies of the seasonal variation of the distribution of *B. pseudomallei* in a rice paddy in northern Laos, we noticed that enrichment cultures often failed to isolate *B. pseudomallei* even when it was isolated from the same sample on solid media ^34^. This led to a formal comparison of the consensus method on Lao soil with other culture and molecular methods. The results of the comparison of the molecular methods with culture on Lao soil and water samples have already been published ^13^, and this paper will focus on a comparison of the performance of the consensus soil method with other methods.

## Methods

### Sample collection

Soil sampling was performed during the dry season (April 2013) in a rice paddy near the village of Ban Nabone, Vientiane Province, Laos (18°22’51.4“N, 102°25’27.8”E, altitude 195 m), as previously described ^13^. In brief, samples were collected at two depths (30 cm and 60 cm) at 50 points within a section of the field previously determined to have the highest positivity rates for *B. pseudomallei* by culture ^34^ (total samples = 100). Written permission to collect the samples was obtained from the village office on the authority of the Director of Mahosot Hospital, but only oral informed consent was obtained from the farmers concerned on the advice of the village office. The samples were placed in sterile plastic bags in an insulated box in the shade and maintained at ambient temperature during transport and subsequent manipulation. Once received in the Lao-Oxford-Mahosot Hospital-Wellcome Trust Research Unit (LOMWRU) laboratory in Vientiane, each soil sample was split into six representative sub-samples using the Japanese slab cake method ^35^. One sub-sample of each sample was then shipped to the Mahidol-Oxford Tropical Medicine Research Unit (MORU) laboratory in Bangkok.

### Sample processing

In order to avoid any variations occurring prior to processing, the processing was co-ordinated between the LOMWRU and MORU laboratories so that all methods started simultaneously. One sub-sample from each sample was processed in one of six ways ( Table 1). Samples were collected within 24 h of each other and subsampling was performed up to ~72 h after collection. Processing of all samples was started on the same day, ~120 h after collection. The methods used were as described in the respective references but are summarised briefly below.

**Table 1.** Methods used to process each of 100 soil samples.

	Method	Description	Reference
i.	Conventional semiquantitative culture (ASH)	100 g soil - Semi-quantitative culture on Ashdown agar in LOMWRU using the method used previously in soil surveys in Laos.	3
ii.	Consensus method (CON-VTE).	10 g soil - Culture by the ‘consensus method’ in LOMWRU using Ashdown agar prepared in LOMWRU.	33
iii.	Consensus method (CON-VTE/BKK).	10 g soil - Culture by the consensus method in LOMWRU, using Ashdown agar prepared in MORU and shipped to Laos (in order to help determine whether differences in media performance or laboratory technique might account for differences in the performance of the consensus method in LOMWRU).	33
iv.	Consensus method (CON-BKK).	10 g soil - Culture by the consensus method performed in MORU	33
v.	Direct PCR (DS/qPCR)	0.5 g soil - PCR following direct DNA extraction from soil in LOMWRU	13
vi.	PCR following enrichment (ES/qPCR)	20 g soil - PCR following broth enrichment culture in LOMWRU	13

***i. Conventional semiquantitative culture (ASH).*** 100 g of each soil sample was added to 100 ml of sterile water and suspended by vigorous agitation. The sample was then left to settle overnight. The following day, 2 × 10 μl, 2 × 100 μl and 1 × 500 μl volumes of the supernatant were inoculated onto Ashdown agar plates (containing Trypticase soy agar with 4% glycerol, crystal violet 5 mg/l, neutral red 50 mg/l and gentamicin 8 mg/l ^34^) and 1 ml into 10 ml TBSS-C50, prepared in MORU (containing threonine-basal salt solution (TBSS) plus colistin 50 mg/l) ^32^. The inoculum was then spread evenly to cover the entire surface of each agar plate, and all cultures were incubated at 40–42°C in air. The TBSS-C50 broth was incubated for 48 h and then 10 μl from the surface was subcultured onto an Ashdown agar plate. Ashdown plates were read on days 2, 3 and 4 of incubation.

***ii, iii and iv. Consensus method (CON-VTE, CON-VTE/BKK and CON-BKK).*** 10 ml TBSS-C50 broth was added to 10 g soil and was vortex-mixed for 30 sec before being incubated at 40–42°C in air for 48 h. The surface of the broth (10 μl) was subcultured onto both an Ashdown plate prepared in LOMWRU and an Ashdown plate prepared in MORU. The plates were incubated at 40–42°C in air and read as above. The same method was used in MORU using only locally prepared media.

A single tube of TBSS-C50 was inoculated with a known *B. pseudomallei* clinical isolate and incubated along with the samples as a positive control for the culture methods.

All suspected isolates were screened by agglutination with a latex agglutination reagent specific for the 200 kDa extracellular polysaccharide of *B. pseudomallei* and tested for susceptibility to co-amoxiclav and resistance to colistin. All presumptive isolates were confirmed as *B. pseudomallei* by qPCR ^36^ and 10% of isolates were also confirmed by API 20NE.

***v and vi. Molecular detection (DS/qPCR and ES/qPCR).*** The molecular methods used in this study were based on the methods of Kaestli *et al*. ^2^ and are described in detail in Knappik *et al.* 2015 ^13^. In brief, DNA was extracted directly from ~0.5 g of soil or after enrichment culture. Enrichment was performed as follows: soil was homogenized in modified Ashdown’s broth, shaken for 2 h at 240 rpm, and then incubated at 37°C for 22 h. The liquid phase was decanted and centrifuged (700 × g, 2 min), and the supernatant was removed and aurintricarboxylic acid was added ^2^. After final centrifugation (45 min, 4,000 × g), DNA was extracted from the pellet. DNA was extracted using the MoBio PowerSoil® DNA Isolation kit ^2^ and 4 µl of soil DNA was used to amplify the orf2 stretch of the TTS1 gene of *B. pseudomallei* ^2, 36^. To reduce the effect of inhibitors, 400 ng/µl of bovine serum albumin (BSA, New England Biolab, USA) was added ^4^.

### Statistical analysis

The sensitivity of each method was defined by comparing yield against the cumulative yield for all six methods and the confidence intervals for sensitivities were estimated by using the ci command in STATA. McNemar’s exact test was used to compare the sensitivity of two methods. Statistical analyses were performed using STATA/MP version 14.2 (College Station, Texas, United States).

## Results and discussion

**Figure 1.**
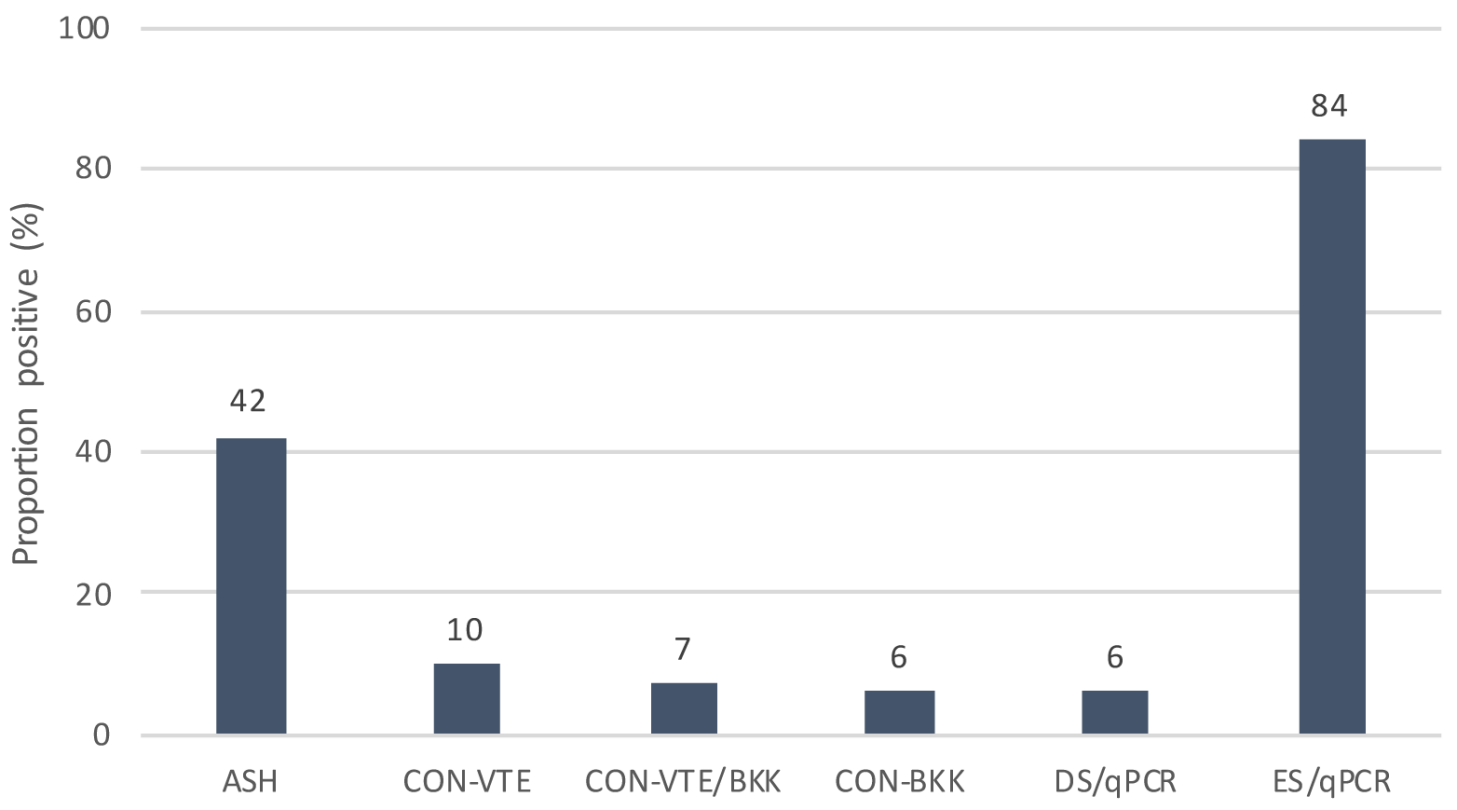
Proportion of samples that yielded *B. pseudomallei* by method. The number of the 100 samples in which *B. pseudomallei* was detected by each method is shown. Abbreviations: ASH, conventional semi-quantitative culture; CON-VTE, consensus method in LOMWRU using locally made media; CON-VTE/BKK, consensus method in LOMWRU using Ashdown agar made in MORU; CON-BKK, consensus method performed in MORU; DS/qPCR, PCR following direct extraction of DNA from soil; ES/qPCR, PCR following broth enrichment culture.

The proportion of the 100 samples in which *B. pseudomallei* was detected by each method is shown in Figure 1. Overall, *B. pseudomallei* was detected in 85 samples by at least one method.

The lowest sensitivities (7% [6/85]; 95% CI: 2.6-14.7%) were obtained using the consensus method in MORU and by PCR following direct extraction of DNA from soil. We have previously reported the low yield of direct DNA extraction from soil ^13^ and this will not be discussed further here. The sensitivity of the consensus method conducted in LOMWRU using locally made media (12% [10/85]; 95%CI 5.8-20.6%) or media prepared in MORU (8% [7/85]; 95%CI 3.4-16.2) was slightly higher than the sensitivity of the consensus method in MORU although this did not achieve significance (p=0.29 and p>0.99, respectively). The sensitivity of the conventional culture method (49% [42/85]; 95%CI 38.4-60.5%) was significantly higher than that of the three consensus methods (all p values<0.001). The sensitivity of PCR following an enrichment culture step (98.8% [84/85]; 95%CI 93.6-99.9) was significantly higher than that of the conventional culture method and all other methods (all p values<0.001). ( Figure 1). There were, however, two samples in which *B. pseudomallei* was not detected by conventional culture but in which it was isolated using one of the consensus methods (in one case only in MORU and in the other in all three variations). There was also a single sample from which *B. pseudomallei* was isolated by conventional culture but in which *B. pseudomallei* was not detected by any other method, including PCR following enrichment culture.

We and others have already demonstrated that PCR following enrichment culture is currently the most sensitive method for the detection of *B. pseudomallei* in both soil and surface water samples ^2, 13, 37^. However, in this study we showed that the consensus method was significantly less sensitive than the conventional culture method when using soil from a field in Ban Nabone, Vientiane Province, Laos, some 560 km away from Ubon Ratchathani in Thailand where the consensus method was originally evaluated and found to have high sensitivity. This difference between the conventional culture method and the consensus method could not be explained by variations in culture media or in the experience of the staff reading the culture plates, as we controlled for all of these factors. The reasons for this variation in the sensitivity of the consensus method on soil from different regions is not known, but could include differences in the numbers of *B. pseudomallei* present in the soil and the amounts of soil processed in the different methods, differences in the range of competing flora present resulting in overgrowth of *B. pseudomallei* in the enrichment broth, differences in the soil type (for example clay as opposed to sandy soil), and possibly the activity of lytic bacteriophages during the enrichment culture step. The different amounts of soil used in the various methods is also likely to influence the sensitivity of each method. Others have also reported finding that *B. pseudomallei* was not isolated from broth cultures despite its apparent enrichment as evidenced by PCR ^37^. The possibility of the organism being in a ‘viable but non-cultivable state’ has been discussed, but this would not explain the apparent amplification of the organism by enrichment culture when comparing the results of direct extraction and PCR with those of enrichment culture and PCR.

There are a number of potential limitations of this study. First, it was conducted in only a single location and it is thus impossible to say how widespread is the issue of sub-optimal sensitivity of the consensus method. However, the fact that it fails to detect a substantial proportion of *B. pseudomallei*-positive samples in at least one location should alert other researchers to this possibility wherever they are working. Secondly, the uneven distribution of *B. pseudomallei* in soil could have accounted for some sub-samples not containing the organism, although we attempted to minimise the risk of this by conducting the sub-sampling using the Japanese slab-cake method. Thirdly, the culture methods are dependent on highly skilled technicians and detection of *B. pseudomallei* depends on them being able to recognise colonies with the appearance of *B. pseudomallei*, meaning that atypical (e.g. moist or mucoid) colonies might be missed, although this is the case with both culture methods. Fourthly, although it is generally agreed that the orf2 stretch of the TTS1 gene is highly specific for *B. pseudomallei*, there may be other as yet uncharacterised organisms closely related to *B. pseudomallei* in the environment that could have given false-positive PCR reactions.

Whilst preliminary and requiring confirmation in other sites, the implication of these findings is that anyone using the consensus method alone might fail to isolate *B. pseudomallei* in a given area, especially if only a small number of samples are tested. Unfortunately, the conventional method is time-consuming, labour-intensive, and requires highly trained and experienced staff to detect small numbers of colonies of *B. pseudomallei* in the midst of a range of competing flora. These results also demonstrated that no method is perfect in detecting *B. pseudomallei* in environmental samples. Despite the higher overall sensitivity of the conventional culture method, there were still two samples from which *B. pseudomallei* was isolated using the consensus method but which were culture-negative by the conventional method, just as there was one sample from which *B. pseudomallei* was isolated by the conventional method despite not being detected by PCR following enrichment.

The development of the consensus method was intended to try to standardise the work being done by many research groups to determine the global distribution of *B. pseudomallei* in the environment ^33^. Although the consensus method has been successful in isolating *B. pseudomallei* from soil in many regions in Thailand ^10^, we believe that it is important that other researchers in this field are made aware that it appears not to have equivalent sensitivity everywhere. Until we understand the reasons why the consensus method has a higher sensitivity in some places than others, we caution others conducting such studies that a failure to isolate *B. pseudomallei* from the environment using the consensus method does not mean that it is not present. Based on our own experience, we suggest that perhaps the most logical approach to looking for *B. pseudomallei* in a new environment would be to use enrichment culture followed by PCR as a screening method, and then to attempt a range of culture methods on PCR-positive samples until one is found that is able to isolate *B. pseudomallei*.

## Data availability

Open Science Framework: Evaluation of consensus method for detection of *B. pseudomallei* in soil, https://doi.org/10.17605/OSF.IO/35GHQ ^38^

Data are available under the terms of the Creative Commons Zero “No rights reserved” data waiver (CC0 1.0 Public domain dedication).
