# Supplementary material for: Evaluation of consensus method for the culture of Burkholderia pseudomallei in soil samples from Laos
Source: Wellcome Open Res. 2018 Nov 21;3:132. Originally published 2018 Oct 15. [Version 2] doi: 10.12688/wellcomeopenres.14851.2 (PMC6283377; doi:10.12688/wellcomeopenres.14851.2)
Supplement: Peer review history

# Peer review: Referee response for version 2

**DOI:** 10.21956/wellcomeopenres.16288.r34299

**Published:** 2018-12-05

**Authors:** Rasana W Sermswan

**Affiliations:** 1 Melioidosis Research Center, Faculty of Medicine, Khon Kaen University (KKU), Khon Kaen, Thailand; 2 Department of Biochemistry, Faculty of Medicine, Khon Kaen University, Khon Kaen, Thailand

No further comment is required. The author responses fulfilled my requirement.

I have read this submission. I believe that I have an appropriate level of expertise to confirm that it is of an acceptable scientific standard.

# Peer review: Referee response for version 1

**DOI:** 10.21956/wellcomeopenres.16180.r34075

**Published:** 2018-11-07

**Authors:** Rasana W Sermswan

**Affiliations:** 1 Melioidosis Research Center, Faculty of Medicine, Khon Kaen University (KKU), Khon Kaen, Thailand; 2 Department of Biochemistry, Faculty of Medicine, Khon Kaen University, Khon Kaen, Thailand

The manuscript compared the performance of the consensus soil method with other methods to determine the distribution of B. pseudomallei in the soil. The authors selected Ban Nabone, Vientiane Province, Laos, some 560 km away from Ubon Ratchathani in Thailand where the consensus method was originally evaluated and found to have high sensitivity as their study area. This area was found to be much less sensitive by the consensus method than the conventional culture method. The authors concluded that no single method is adequate to conclude any environment to have or not have B. pseudomallei. The conventional culture method should be applied in a new environment.

 My comments are as follows:

 1. The samples were collected at two depths (30 cm and 60 cm) at 50 points to the total of 100 samples. 

 Even though the aim of this manuscript is to compare methods of detection of B. pseudomallei in the environment, however, results and discussions of other points of the study should be beneficial and worth to describe.

 What are the results from the different depth? Are they similar by all methods or different? Does the bacterium found equally in 2 depths?

 2. Why was the consensus method that was applied in the same area with high sensitivity in the previous report found to be much less sensitive than conventional culture method in this study?

 3. The authors discussed that the difference between the conventional culture method and the consensus method could not be explained by variations in culture media or in the experience of the staff reading the culture plates, as they controlled for all of these factors. 

 Could it be the soil type that makes them different? Conventional culture method mixed 100g of soil with 100ml water and incubated overnight before spreading on Ashdown medium while consensus method mixed the soil with water and the spread. Conventional culture, therefore, may be better for clay soil that holds bacterium stronger than sandy soil. Analysis of the soil types in this study may provide some clue. This may also help predicted suitable method for environmental study in new areas.

 4. All methods used a different amount of soil. A hundred grams of soil used in conventional should be better than 10 gram. Does 100g or 10g used in conventional culture give similar sensitivity? The authors also mention that DNA extracted from 0.5g soil and used for PCR detection is not sensitive. However, 84 positives by 20g soil with enrichment method may be too high? How could the author make sure that there was no contamination or carryover of positive soil to the negative soil during DNA extraction or high sensitivity PCR amplification?

 Minor comment:

 Results and discussion

 "There were, however, two samples in which B. pseudomallei was not detected by conventional culture but in which it was isolated using one of the consensus methods (in one case only in MORU and in the other in all three variations)." 

 This sentence may need be rewritten to make it more understandable.

I have read this submission. I believe that I have an appropriate level of expertise to confirm that it is of an acceptable scientific standard, however I have significant reservations, as outlined above.

# Response

**Published:** 2018-11-19

**Authors:** David A. B. Dance

**Affiliations:** Lao-Oxford-Mahosot Hospital-Wellcome Trust Research Unit, Lao People's Democratic Republic

*We are grateful to the referees for taking the time to read and comment on our manuscript, which has enabled us to make improvements in Version 2.  Our responses are given in italics below.*

**Referee 2**

The manuscript compared the performance of the consensus soil method with other methods to determine the distribution of B. pseudomallei in the soil. The authors selected Ban Nabone, Vientiane Province, Laos, some 560 km away from Ubon Ratchathani in Thailand where the consensus method was originally evaluated and found to have high sensitivity as their study area. This area was found to be much less sensitive by the consensus method than the conventional culture method. The authors concluded that no single method is adequate to conclude any environment to have or not have B. pseudomallei. The conventional culture method should be applied in a new environment.

My comments are as follows:

1. The samples were collected at two depths (30 cm and 60 cm) at 50 points to the total of 100 samples. 

Even though the aim of this manuscript is to compare methods of detection of B. pseudomallei in the environment, however, results and discussions of other points of the study should be beneficial and worth to describe.

What are the results from the different depth? Are they similar by all methods or different? Does the bacterium found equally in 2 depths?

*As the referee says, the purpose of this study was to compare the yield of the different methods and we feel that to include a discussion of this aspect would distract attention from the key message.  For further details about the yield from different soil depths, readers are referred to Manivanh L, Pierret A, Rattanavong S, Kounnavongsa O, Buisson Y, Elliott I, et al. Burkholderia pseudomallei in a lowland rice paddy: seasonal changes and influence of soil depth and physico-chemical properties. Scientific Reports. 2017;7(1):3031, and to the supplementary data of this paper.*

2. Why was the consensus method that was applied in the same area with high sensitivity in the previous report found to be much less sensitive than conventional culture method in this study?

*The reason for the lower yield of the consensus method in Ban Nabone is not known at this stage, but some possible explanations are explored in paragraph 4 of the ‘Results and discussion’ section.*

3. The authors discussed that the difference between the conventional culture method and the consensus method could not be explained by variations in culture media or in the experience of the staff reading the culture plates, as they controlled for all of these factors. 

Could it be the soil type that makes them different? Conventional culture method mixed 100g of soil with 100ml water and incubated overnight before spreading on Ashdown medium while consensus method mixed the soil with water and the spread. Conventional culture, therefore, may be better for clay soil that holds bacterium stronger than sandy soil. Analysis of the soil types in this study may provide some clue. This may also help predicted suitable method for environmental study in new areas.

*We agree that this is another possibility and have added the phrase “difference in the soil type” to the section referred to above.*

4. All methods used a different amount of soil. A hundred grams of soil used in conventional should be better than 10 gram. Does 100g or 10g used in conventional culture give similar sensitivity? The authors also mention that DNA extracted from 0.5g soil and used for PCR detection is not sensitive. However, 84 positives by 20g soil with enrichment method may be too high? How could the author make sure that there was no contamination or carryover of positive soil to the negative soil during DNA extraction or high sensitivity PCR amplification?

*Again, we agree that the different amounts of soil processed in the different methods could be an important factor and have added “and the amounts of soil processed in the different methods” into the section referred to above.  We accept that contamination is always a potential problem with PCR methods, and we used a standard approach to minimise the risk of this by using physically separated rooms for DNA extraction, PCR preparation and DNA addition. Furthermore, no template controls were always negative.  In addition, this would not account for the differences between the results obtained with DS/qPCR and ES/qPCR.*

Minor comment:

Results and discussion

"There were, however, two samples in which B. pseudomallei was not detected by conventional culture but in which it was isolated using one of the consensus methods (in one case only in MORU and in the other in all three variations)." 

This sentence may need be rewritten to make it more understandable.

*We realise that this sentence is complex but we believe that it clearly describes the results obtained.*

# Peer review: Referee response for version 1

**DOI:** 10.21956/wellcomeopenres.16180.r34076

**Published:** 2018-10-25

**Authors:** Ya-Lei Chen

**Affiliations:** 1 Department of Biotechnology, National Kaohsiung Normal University, Kaohsiung, Taiwan

This manuscript is valuable to remind us that the isolation strategy for *B. pseudomallei* in the environment should strictly consider its own physiochemical parameters. Consensus methodology has shown successful isolation of *B. pseudomallei* from Thai soil but, in this report, it shows a worse isolation from Lao soil. I believe that *B. pseudomallei* strains exhibit many genomovars, morphovars and even pathovars in different areas that are endemic to melioidosis because B. pseudomallei accommodate in different soil condition through a long-term evolution by geographical seclusion. Thus, different isolation methods used in different places are reasonable. However, several concerns have to be considered:

 1. Even if the soil was collected from same place, it was very difficult to ensure that presence or absence of *B. pseudomallei *or any organisms that were antagonistic to *B. pseudomallei* can be found in different pieces (sub-sample). Actually, the distribution of *B. pseudomallei* in soil sample is very uneven.

 2. The suspect isolates were chosen following the colony morphology from consensus and ASH methods. However, this technique is experience-dependent because *B. pseudomallei* colony morphovars have been widely reported in these media. Several atypical morphologies such as smaller, moisturized or mucoid could appear in environmental isolates but they probably were ignored and seldom found in clinical isolates, usually appearing a dry and winkled colony morphology.

 3. The fragment of *orf2* stretch of the TTSS could be amplified by soil organisms that are not related to *B. pseudomallei*. In other words, false positive probably existed in ES/qPCR method.

 4. In Fig. 1, the proportion of positive sample in ES/qPCR is 84 or 85?

 5. In the statistical analysis, there is no mention of how to calculate 95% CI.

 Overall, I agree with the authors’ conclusion that *B. pseudomallei* in a new environment would be to use enrichment culture followed by PCR as a screening method. Repeatedly isolation for *B. pseudomallei* by selective media from PCR positive sample is needed.

I have read this submission. I believe that I have an appropriate level of expertise to confirm that it is of an acceptable scientific standard.

# Response

**Published:** 2018-11-16

**Authors:** David A. B. Dance

**Affiliations:** Lao-Oxford-Mahosot Hospital-Wellcome Trust Research Unit, Lao People's Democratic Republic

Evaluation of consensus method for the culture of *Burkholderia pseudomallei* in soil samples from Laos

**Response to Referees’ Comments**

*We are grateful to the referees for taking the time to read and comment on our manuscript, which has enabled us to make improvements in Version 2.  Our responses are given in italics below.*

**Referee 1**

This manuscript is valuable to remind us that the isolation strategy for *B. pseudomallei* in the environment should strictly consider its own physiochemical parameters. Consensus methodology has shown successful isolation of *B. pseudomallei* from Thai soil but, in this report, it shows a worse isolation from Lao soil. I believe that *B. pseudomallei* strains exhibit many genomovars, morphovars and even pathovars in different areas that are endemic to melioidosis because B. pseudomallei accommodate in different soil condition through a long-term evolution by geographical seclusion. Thus, different isolation methods used in different places are reasonable. However, several concerns have to be considered:

1. Even if the soil was collected from same place, it was very difficult to ensure that presence or absence of *B. pseudomallei *or any organisms that were antagonistic to *B. pseudomallei* can be found in different pieces (sub-sample). Actually, the distribution of *B. pseudomallei* in soil sample is very uneven.

*We agree with the referee about this, which is why we went to considerable lengths to ensure that the sub-samples were as representative as possible by using the ‘2D Japanese slab-cake’ method, as mentioned in the ‘Methods’ section.  We have added a paragraph in the ‘Results and discussion’ section to address this and other limitations of the study raised by the referees.*

2. The suspect isolates were chosen following the colony morphology from consensus and ASH methods. However, this technique is experience-dependent because *B. pseudomallei* colony morphovars have been widely reported in these media. Several atypical morphologies such as smaller, moisturized or mucoid could appear in environmental isolates but they probably were ignored and seldom found in clinical isolates, usually appearing a dry and winkled colony morphology.

*Again, we agree about this and have included this in the discussion of the limitations referred to above.*

3. The fragment of *orf2* stretch of the TTSS could be amplified by soil organisms that are not related to *B. pseudomallei*. In other words, false positive probably existed in ES/qPCR method.

*We are not clear of the referee’s evidence for this.  This target has been widely used by others for the detection of B. pseudomallei in both clinical and environmental samples and is generally believed to be highly specific. However, since we accept that there are probably ‘near-neighbours’ of B. pseudomallei in the environment that have not yet been fully characterised, we have included this in the paragraph on limitations as above.*

4. In Fig. 1, the proportion of positive sample in ES/qPCR is 84 or 85?

*This was 84 of the 85 samples positive by any method as shown in the figure, the discrepancy being the result of the single sample that was negative by ES/qPCR but positive by conventional culture.*

5. In the statistical analysis, there is no mention of how to calculate 95% CI.

*We have added a statement in the ‘Statistical analysis’ section that says “The confidence intervals for sensitivities were estimated by using the ci command in STATA”.*

Overall, I agree with the authors’ conclusion that *B. pseudomallei* in a new environment would be to use enrichment culture followed by PCR as a screening method. Repeatedly isolation for *B. pseudomallei* by selective media from PCR positive sample is needed.
